# Fast Degaussing Procedure for a Magnetically Shielded Room

**DOI:** 10.3390/ma17235877

**Published:** 2024-11-30

**Authors:** Peter A. Koss, Jens Voigt, Ronja Rasser, Allard Schnabel

**Affiliations:** 1 Fraunhofer-Institut für Physikalische Messtechnik (IPM), Georges-Koehler-Allee 301, 79110 Freiburg, Germany; 2 Physikalisch-Technische Bundesanstalt (PTB), Abbestr. 2, 10587 Berlin, Germany

**Keywords:** degaussing, residual magnetic field, magnetic shielding, OPM, MEG

## Abstract

A demagnetization study was conducted on a magnetically shielded room (MSR) at Fraunhofer IPM, designed for applications such as magnetoencephalography (MEG) and material testing. With a composite of two layers of mu-metal and an intermediate aluminum layer, the MSR must provide a residual field under 5 nT for the successful operation of optically pumped magnetometers (OPMs). The degaussing process, employing six individual coils, reached the necessary residual magnetic field within the central 1 m^3^ volume in under four minutes. Due to the low-frequency shielding factor of 100, the obtained average residual field is shown to be limited by environmental residual field changes after degaussing and not by the degaussing procedure.

## 1. Introduction

Magnetically shielded rooms (MSRs) are specialized enclosures designed to create controlled environments with reduced residual magnetic fields and minimized influence from external magnetic disturbances occurring nearby. MSRs are mainly used for bio-magnetic measurements in neuroscience, e.g., [1,2], and in fundamental physics experiments, e.g., [3,4].

Research efforts have focused on achieving low residual magnetic fields and field gradients in MSRs to enable high-resolution fundamental experiments and the use of sensitive magnetic sensors [5,6]. Practical medical applications must satisfy additional factors like site restrictions, the overall cost per measurement, preparation and measuring time, and patient’s comfort. Meanwhile, sensitive and lightweight magnetic field detector systems have been introduced, leading to the development of wearable magnetoencephalography (MEG) systems [2]. These advancements enable patients to move during measurements or even walk within the MSR [7,8]. Suitable magnetic field detectors for such applications are optically pumped magnetometers (OPMs). For the optimal operation of OPMs in spin-exchange relaxation-free (SERF)-mode, an absolute magnetic field of <5 nT is required. It has been reported that such conditions can be obtained within the central 1 m^3^ of a two-layer MSR [5,9]. 

Fraunhofer IPM in Freiburg, Germany, purchased a standard 2 + 1-layer MSR from VAC, Germany. The MSR consists of a two-layer mu-metal for low-frequency shielding and one eddy current layer (aluminum) for high frequency shielding [10]: thus, the nomenclature 2 + 1 layer MSR. The MSR walls incorporate degaussing coils. Every time the MSR is opened, the MSR, and especially its door, is magnetized by the external magnetic field, e.g., the earth magnetic field. One of the challenges is to shorten the degaussing procedure so that the MSR can be degaussed in an acceptable time after a new person enters. 

While several studies have documented the capabilities of MSRs in achieving low residual fields, a distinguishing factor of our work is the emphasis on a rapid degaussing, which could be performed after each door operation to obtain reproducible, low, and stable field conditions. This requirement has not been explicitly addressed in the previous literature, such as the works cited in [5,9,11,12]. By incorporating techniques such as a degaussing configuration, which allows simultaneous degaussing across all axes, we demonstrate that reproducible residual fields well below 5 nT can be achieved within four minutes, enhancing the practicality and efficiency of MSR use for ongoing experimental applications. The subsequent sections detail the methods and results, reflecting the effectiveness of our approach in ensuring reliable and stable low-field environments. 

## 2. Materials and Methods

**Figure 1:**
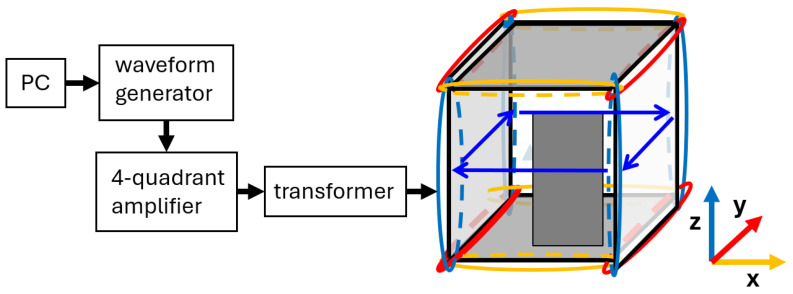
Schematic illustration of the experiment’s layout established to degauss the MSR. The identically colored coils on the edges are interconnected in a series so that the magnetic field induces a complete magnetic loop inside the shielding material around the inner volume, as indicated by the blue arrows for the blue coils.

The MSR at Fraunhofer IPM is a cube where the shells have the following dimensions: the internal mu-metal shell has a 2.57 m side length (3 mm thick mu-metal), the aluminum shell has a 2.60 m side length (8 mm thick aluminum), and the outer shell has a 2.91 m side length (3 mm thick mu-metal). The door is placed in the front-right part of the cube, the gray rectangle in Figure 1, and it has dimensions of 1 m × 2 m. Three pairs of Helmholtz-like coils surround the MSR such that fields can be applied. Their dimensions are approximately 2.9 m × 2.9 m with a spacing between them of 2.9 m. 

The MSR is equipped with six autonomous degaussing coils, with three coils for each of the two mu-metal shells. The three coils for one shell are depicted in Figure 1. Each degaussing coil consists of four individual coils positioned at the cube’s edges along one of the three MSR axes, which are connected in series to form a large toroidal coil. Each coil on the edge of the MSR has 5 windings of 2.5 mm^2^ Cu-wire. The z-coil generated a magnetic flux within the shielding material on a plane orthogonal to the z-axis, as shown in Figure 1. When the coil for only one axis of one shell is activated, it is referred to as an I-configuration (one color). The DC resistance of each I-coil (I_x_, I_y_ and I_z_) is about 1 Ω. If the coils for two axes of one shell are operated simultaneously as introduced in [9], it is called an L-configuration (two colors). Operating the coils for all three axes of one shell simultaneously is a Z-configuration (all three colors). The number of “strokes” in the representation with letters, I, L, and Z, corresponds to the number of coils being combined. The sequential arrangement of the strokes reflects the series connections of the coils, while the angles between the strokes indicate the different field directions of the combined coils.

The degaussing signal for operating the coils was generated as illustrated in Figure 1. A PC-programmed waveform generator equipped with a 16-bit analog output card from National Instruments produced the degaussing waveform, a linearly declining sinusoid that comprised 1500 periods at a frequency of 7 Hz. Prior to the amplitude decrease, the amplitude was linearly increased to its maximum over 10 periods, followed by 10 periods at the maximum amplitude to stabilize conditions. Each complete degaussing cycle takes 3 min and 40 s. Additional time is needed at the beginning, between different degaussing cycles, and at the end to disconnect, change, and connect the cables. The degaussing waveform was fed into a four-quadrant amplifier (Rohrer GmbH, Munich, Germany) to convert the voltage-driven signal into an oscillatory current-driven signal with a peak amplitude of approximately 20 A. This alternating current was then passed through a 7 Hz transformer to remove any residual DC offsets before being applied to the selected degaussing coils within the MSR walls. The transformation ratio used was 1:1, but it can be used to adapt the amplifier values V and I to the resistance of the degaussing coil.

**Figure 2:**
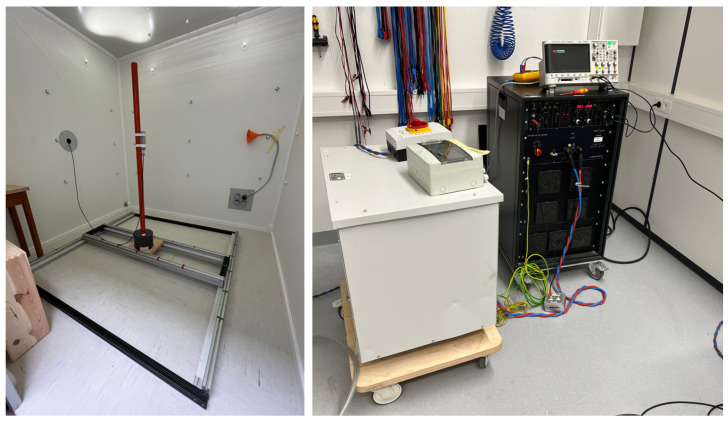
Images of the experimental setup elements: On the left: a picture of the mapping framework using an aluminum rail to adjust the xy-position of the pole, allowing the fluxgate to be positioned at different z-positions. On the right: a photo of the four-quadrant amplifier (black) that drives the degaussing current through the degaussing coil and the 7 Hz transformer (large white box) to eliminate DC offsets.

The MSR was demagnetized by applying degaussing fields in multiple configurations, followed by mapping the residual field in a central 1 m^3^ volume using a 3-axis Bartington fluxgate (MAG03 MCL 70, Witney, UK) for a 3D visualization of the residual field distribution. Measurements were taken on a 25 cm grid at 125 positions (5 × 5 × 5). Figure 2 shows the equipment used for degaussing the MSR and mapping the field. The field scan was manually performed by a non-magnetic person inside the MSR and was completed within 26 min. To minimize systematic uncertainty in the static residual field measurement, the offset was measured before and after a full scan, which took an additional 3 min. Alongside the fluxgate inside the MSR, readings from an exterior reference fluxgate (MAG03 MC 1000) were also recorded. 

Initially, we conducted an inspection of the residual field in the MSR using a small, single-channel fluxgate mounted on a wooden rod, which was inserted into the MSR through a feedthrough hole. Our objective was to verify that the sequential degaussing procedure for the I_x_-, I_y_-, and I_z_-degaussing coils on both the outer and inner layers effectively reduces the residual field. This method was first reported in [5]. Subsequently, three configurations for fast degaussing were tested with a person inside the MSR who scanned the field distribution post-degaussing. The three configurations are as follows:

1. **posI**: Interconnecting the I-configuration of both shells in series, instead of operating them individually in sequence, which halves the degaussing time. The coils were connected such that the magnetic fields in the inner and outer shells point in the same direction.
2. **negI**: Replicating the first configuration but reversing the polarity. While this does not reduce the degaussing time compared to the **posI** configuration, it helps evaluate the reproducibility and imperfections of the degaussing function. A rapidly decreasing demagnetization function or a DC offset in the demagnetization current can create field contributions near the MSR center point, resembling a curl that reverses direction with polarity changes. Additional details are available in Appendix B.
3. **posZ**: Interconnecting all coils—across both shells and in all three directions—in series. This allows for the complete degaussing of the MSR in a single operation. The degaussing time was reduced by another factor of three, making it six times faster and eliminating the additional time to change the connections to different degaussing coils. Note that “**posZ**” indicates the connection of the three I-coils, not the geometric axis.

To better understand the impact of door operations, we included multiple sequences of opening and closing the door to record the corresponding field changes after every measurement of a 3D field map. Observations at the center of the MSR indicated that these field changes were inconsistent and were significantly influenced by both the length of time the door was open and the angle at which it was opened. However, the absolute value increase observed at the MSR center was on the order of 5 nT. These findings confirmed our assumption that degaussing after each door operation is necessary to achieve reproducible and stable measurement conditions, maintaining a residual field below 5 nT within the central 1 m^3^ of the MSR.

During the measurement campaign, the shielding factor (SF) of the MSR was measured along three spatial directions to aid in interpreting the residual field maps. A set of three Helmholtz-like coil systems, permanently installed outside the MSR, were used to apply the excitation field. The same apparatus, as shown in Figure 1, was used to drive the coils, except for the transformer. Additionally, an automated switch controlled by the PC was incorporated to toggle between the Helmholtz coils in different directions. This setup enabled automated data acquisition at night, which is crucial as low-frequency measurements require extended measurement times. 

## 3. Results

**Figure 3:**
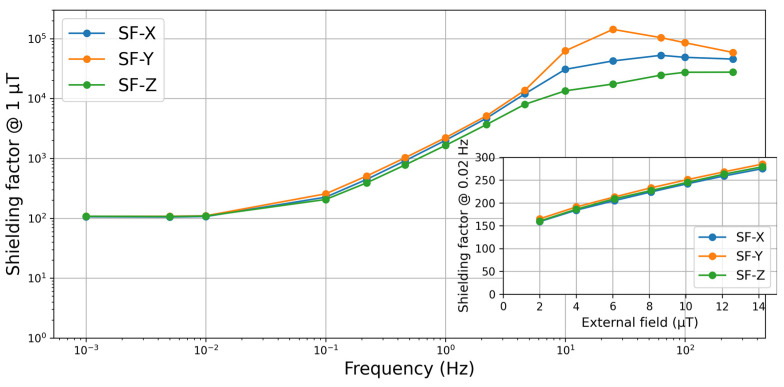
Measured shielding factor (SF) curves for the three axes of the two-layer MSR with a 1µT effective excitation field strength. In the inset: dependence of the SF at 0.02 Hz on the amplitude of the excitation field.

The measured shielding factor curve (Figure 3) is typical for this type of MSR. 

The low-frequency shielding factor for (f → 0), known as the quasi-static shielding factor, is approximately 100 in all three MSR directions. Considering ongoing discussions about the specifications of the measurement conditions, we also examined the dependence of the SF at 0.02 Hz on the applied excitation amplitude, as shown in Figure 3. Within the measured amplitude range, the SF increases almost linearly, with a slight reduction in slope at larger amplitudes. This dependence is strong enough that the measurement conditions should be clearly specified when purchasing an MSR and when calculating expected damping factors from SF curves or values provided by the manufacturer.

The environmental field changes measured before the MSR construction at the planned MSR site were 0.2 µT in a 1 h time interval during daytime. With a low-frequency shielding factor of 100, we expected up to 2 nT field changes during the residual field measurements. To obtain an accuracy of below 1 nT we had to provide a possibility to correct for such external changes and for DC-offset drifts on the recorded data channels in the data acquisition system. Therefore, we installed a reference fluxgate next to the MSR and determined the overall DC offsets (including the data acquisition system) for the three fluxgate channels inside the MSR before and after a field scan. The average value of both DC offsets is subtracted from the raw data of the respective channel to take care of the constant portion of the overall DC offset. The magnetic field at the center point of the MSR was measured seven times, i.e., before and after the scan, when we changed to the next layer with 25 points, and the measurement of the center during the scan itself. The additional measurements allowed us to reduce the influence of outside field changes and DC-offset drifts of the measurement setup. The details of the procedure to obtain the “corrected” data from the “uncorrected” data are reported in Appendix A.

**Figure 4:**
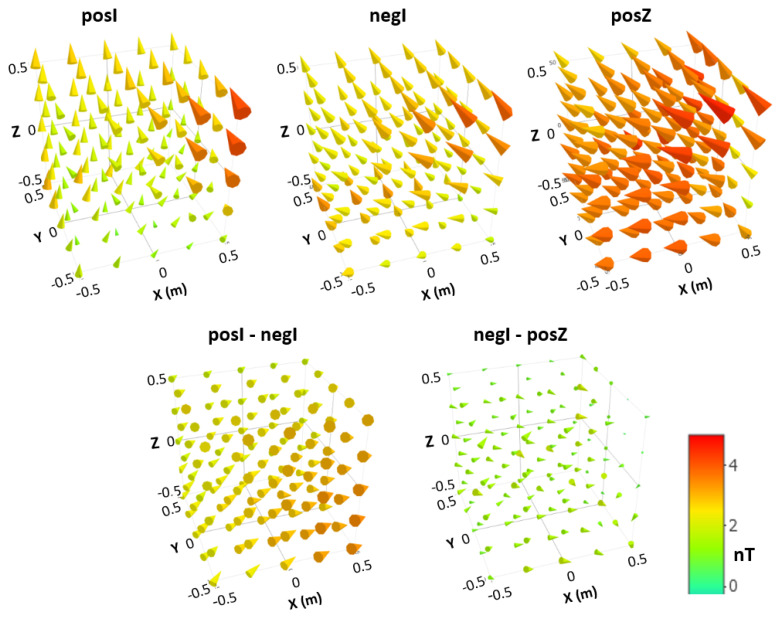
Illustration of the measured 3D field distributions for the three different degaussing configurations: **posI**, **negI**, and **posZ** (top row). Below, the difference between the respective 3D maps is shown. All maps use the same color code. Table 1a provides characteristic values for these 3D field illustrations. Note that the scaling of the height of the cones relative to the absolute field value reveals minor differences in the maximum amplitude, attributed to a much larger base surface. The difference between **negI** and **posZ** is minimal because both fields have a similar direction, whereas **posI** and **negI** exhibit slightly different directions.

We calculated the difference between the size of the 125 field vectors calculated from the corrected and uncorrected data. The differences are always in the range of ±0.5 nT. The average is below 0.05 nT, indicating that there is no shift in the field in one direction. The corrections are large enough to reduce any inconsistencies observed in the original sequentially measured data, i.e., large local jumps of the measured values, between adjacent data points in different layers, which were not measured shortly after each other. The correction is so successful that such field jumps due to outside field changes are reduced to a level that is hard to recognize in the corrected data, presented here as 3D field distributions in Figure 4. Table 1a provides characteristic values for the shown 3D field maps and map differences. 

The linear drift corrected is usually below ±0.2 nT for the timespan to measure the 125 points. This is on the order of the observed difference in the fluxgate DC-offset measured before and after the 3D field scan. An exception was the x-channel for the first field scan (**negI**), where the correction is ±0.45 nT. We think that other DC-offset contributions of the data acquisition still dominated after switching on the equipment. 

The accuracy of the fluxgate DC-offset determination of ±0.1 nT is one contribution to the uncertainty of the field measurement. Additionally, the measurement is still limited by the remaining field changes, which could not be accounted for in the correction (residuals of the fit explained in Appendix A). The standard deviation for the seven repeated measurements of the absolute value of the corrected magnetic field vector B at the MSR center is the remaining statistical accuracy of the measurement. It is for **posI** 0.16 nT (before correction 0.24 nT), for **negI** 0.17 nT (0.35 nT), and for **posZ** 0.065 nT (0.095 nT).

It was interesting to find that combining corresponding outer and inner degaussing coils as well as combining all degaussing coils lead to field values below 5 nT within the 1 m^3^ central volume, even without corrections for external disturbances and apparatus DC-offset drifts. For the **posZ** configuration, where all six degaussing coils are connected in series, the duration of the degaussing sequence, used only once, is 3 min and 40 s. By employing PC-operated switches controlled by the PC shown in Figure 1, to manage the connection and disconnection of the degaussing coils, the entire demagnetization process can be completed in less than 4 min once the MSR door is closed.

## 4. Discussion

The improvement on the measured 3D field maps by the correction allows us to analyze smaller effects on the measured residual field distributions.

The degaussing with reversed polarity of the degaussing coil (**posI** and **negI**) connection was performed to check the influence of the used degaussing function. A DC offset on the applied degaussing function and an insufficient number of active AC cycles of the degaussing function will cause a remaining magnetic field signature, which looks like a field around the MSR center. This field would increase in amplitude towards the MSR walls. This curl-like structure changes direction when the sign of the applied degaussing function is reversed (an explanation for the curl is provided in Appendix B). Therefore, calculating **posI-negI** will enhance the visibility of this effect and will suppress common field distributions that would indicate non-degaussed areas or magnetic objects inside the MSR. 

There is no visible curl-like structure in **posI-negI** in Figure 4. It is therefore concluded that the DC-offset suppression on the AC degaussing current and the number of degaussing cycles with respect to the maximal current used is sufficient. This would allow us to analyze in further measurements if the degaussing time could be reduced even further by reducing the number of degaussing cycles. 

In Figure 4**,** the strongest field is always at the top right corner and is not seen in the difference. We concluded that there is a magnetic field source in the top right area of the door that is not changed by the degaussing. We checked that the door does not have a larger door gap at the top right corner after closing. After ruling out this possible source, we checked the door area with a handheld fluxgate (Fluxmaster, Stefan Mayer Instruments, Dinslaken/Germany). There, we found overly magnetic stainless-steel screws (A4) located in the MSR door, particularly one near the top right corner. Based on the measurements, we replaced the A4 screws with titanium screws. This effectively eliminated the dominant contribution to the residual field, as confirmed by repeating the measurement around the door using the handheld fluxgate. 

The **negI-posZ** difference in Figure 4 is zero within the measurement uncertainty. **posI-negI,** on the other hand, shows field vectors in one direction with increasing amplitude towards the door. This is an expected field change if the surrounding field of the MSR is different at the time of the 3D field map. The increase in the direction of the door can be explained by the expected poorer shielding factor at the door position. However, it could also be due to a different environmental field during degaussing.

Degaussing aims to remove all previous magnetization of the shielding material of the MSR, and it equilibrates the MSR to the magnetic field conditions at the MSR site. This equilibration of the MSR reduces the static magnetic field inside the MSR far below the expected field change by applying the external field to an MSR degaussed in a zero-field environment. The validity of this theoretical prediction was confirmed for a similar MSR by residual field measurements [5] in a larger volume, and the theoretical limit is discussed in [9]. The maximum observed residual field after degaussing at the MSR’s center (see Table 1a) is 3.4 nT and at least a factor of 12,000 lower than the surrounding environmental field of 41 µT measured at the MSR site before the MSR installation. Therefore, even a strong hypothetical environmental field difference of 1 µT during different degaussing attempts would result in a center field difference of less than 83 pT, which is already beyond the resolution of the fluxgate. This effectively rules out the influence of the surrounding field during degaussing as the cause of the field difference **posI-negI** shown in Figure 4. A reasonable explanation is the environmental field change between degaussing and the field scan. This environmental field change is only shielded by the low-frequency shielding factor of about 100. **posI** and **negI** show the same residual field with added different field changes due to different field changes after degaussing.

**Figure 5:**
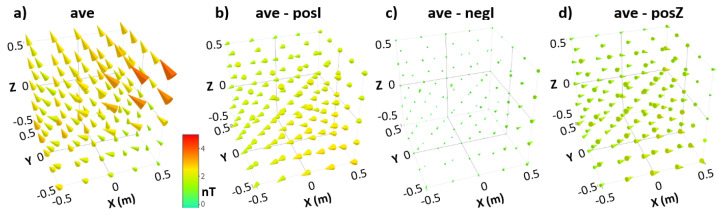
Average field map (**ave**) and the differences between it and the maps **posI**, **negI**, and **posZ**. All maps share the same color code. Table 1b provides characteristic values for these 3D field maps.

Because we already concluded that the residual field contained in **posI** and **negI** and that **negI** and **posZ** are identical within the measurement uncertainty, the three measured 3D field maps show the same residual field distribution. The maps are not identical; they differ due to variations in the field change that occurred after degaussing. The average (**ave**) shown in Figure 5 over all three 3D maps measured is therefore a statistically more accurate measurement of the residual field distribution after degaussing. Figure 5 also shows the differences in the measured 3D field maps to the average. They illustrate the field changes due to the different environmental field at the MSR site during degaussing and the subsequent 3D scan of the field inside the MSR. Characteristic values for these 3D field maps can be found in Table 1b.

The maximum field value of the average 3D map in Table 1b is 3.8 nT, indicating that the residual field after degaussing is below 4 nT. This residual field is primarily influenced by the overly magnetic screws at the door, as previously discussed.

Field distributions common to all three 3D scans contributing to the average, such as the field from the A4 screws at the front right corner and the common residual field, are not present when calculating the differences to the average map. The 3D field map, **ave-negI**, shows that **negI** is very close to the average. The only non-statistically distributed field appears near the door, being just 1 nT larger in the direction of the door hinge. The other two differences, **ave-posI** and **ave-posZ**, display field vectors in a single direction, with amplitude increasing towards the door as expected for an environmental field change after degaussing.

The field structure in ave in the bottom left backside corner of the scan volume at (x, y, z) = (−0.5, 0.5, −0.5) is not visible in all three differences. It is therefore contained in all three 3D scans. This indicates another magnetic field source that is significantly weaker than the screws on the door and whose strength is not affected by demagnetization. 

**Figure A1:**
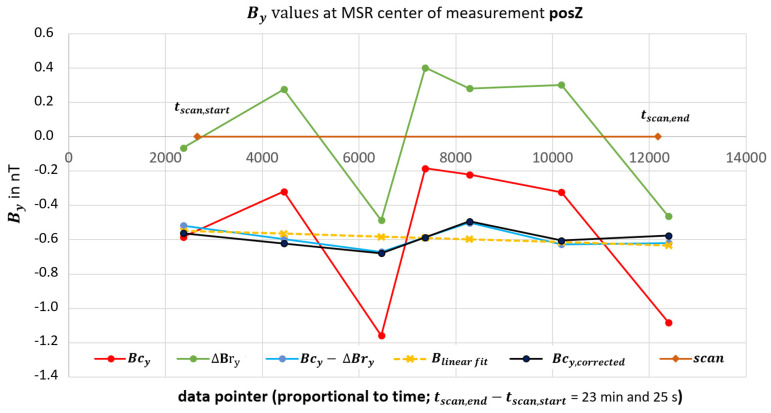
Illustration of the corrections performed to the measured data (red) based on a simultaneous field measurement with an outside reference fluxgate (green) and the repeated measurement of the magnetic field at the center of the MSR at 7 different times. Blue is the difference between the red and the green curve. The yellow line is the calculated linear fit curve to the blue curve. The black dots are the 7 values of the magnetic field at the MSR center after all corrections are applied.

We hypothesize that the difference in the environmental field at the MSR site for the different degaussing times for the different scans caused the largest difference in the measured residual field. As illustrated in Figure A1 of Appendix A, there is a correlation between the environmental field and repeated measurements at the center of the MSR. However, since we did not record the field outside the MSR with the reference fluxgate after degaussing, we cannot confirm that the average field value of the maps (b), (c) and (d) of Figure 5 listed in Table 1b correlate with the change in the environmental field from the time of degaussing to when the 3D scan of the inside field was conducted. 

An active shield that is switched on immediately after degaussing will decrease the measurement uncertainty for the 3D field scans and improve the reproducibility for repeated 3D field measurements. Additionally, it could maintain temporal field stability already during degaussing if the bandwidth of the compensation system is restricted to well below the degaussing frequency of 7 Hz using a low-pass filter incorporated into the feedback loop. Improving the accuracy of a single field map regarding the residual field after degaussing will eliminate the need to average repeated 3D field maps with the same parameter settings. This enhancement is essential for determining whether the degaussing time can be reduced with a shorter degaussing function.

## 5. Conclusions

The two-layer VAC-MSR at Fraunhofer IPM in Freiburg, Germany, which features a low-frequency shielding factor of 100, underwent degaussing processes using different coil configurations that achieved a short degaussing time. This procedure demonstrated that less than four minutes is sufficient to degauss the MSR after it has been opened and subsequently closed for the next measurement. This process successfully achieved a residual magnetic field of less than 5 nT in the central 1 m^3^ and maintained stable field conditions suitable for the operation of SERF-OPMs.

The limitation for the field inside the MSR is not governed by the environmental magnetic field during the degaussing process but rather by changes in the environmental field at the MSR location post-degaussing, which are only attenuated by a shielding factor of 100. Future enhancements will involve the use of active shielding by utilizing the SF-test coils permanently installed on the MSR to further dampen the observed magnetic field changes at the MSR site.

To achieve significantly lower residual fields, it is crucial to avoid using even slightly magnetic materials inside the MSR. Implementing a more robust overall shielding strategy is fundamental. This can be accomplished through active shielding and the removal of overly magnetic components. With these improvements, it is anticipated that a reproducible residual field of below 1.5 nT in the central cubic meter can be attained as demonstrated in [5] for a similar MSR under nearly ideal measurement conditions in the middle of the night. This goal will ultimately depend on the coil configurations and the degaussing function of the MSR.

## Figures and Tables

**Table 1:** List of characteristic values for the 3D field maps: (**a**) for Figure 4, and (**b**) for Figure 5.

(a)						(b)				
Values in nT	posI	negI	posZ	posI-negI	negI-posZ	Values in nT	ave	ave-posI	ave-negI	ave-posZ
**max value**	4.1	3.8	4.6	3.4	2.1	**max value**	3.8	2.6	1.2	2.1
**min value**	0.9	1.6	1.1	1.8	0.3	**min value**	1.3	1.3	0.2	0.7
**mean value**	2.1	2.5	3.5	2.5	1.2	**mean value**	2.3	2.0	0.6	1.6
**STD**	0.6	0.4	0.4	0.4	0.3	**STD**	0.4	0.3	0.2	0.3
**MSR center**	1.9	2.3	3.4	2.5	1.2	**MSR center**	2.1	2.0	0.6	0.0

## Data Availability

The original contributions presented in the study are included in the article, further inquiries can be directed to the corresponding author.

## Appendix A. Correction to the Measured Data

Plotting the seven values measured for the center point Bc_n_ versus time together with the measured outside reference field value Br, a correlation is observed as illustrated in Figure A1. The red curve shows the measured values inside the MSR, and the green curve shows the corresponding field changes outside the MSR. The values are shown with respect to the average outside field during the timespan in which the 125 points of the respective 3D field maps were measured. This time duration is indicated in Figure A1 and labeled with “scan”. The difference between the inside and outside field is shown in blue where the outside field is already scaled to match the inside field changes.

In this case, only a small linear drift shown in yellow is removed from the blue curve to obtain the final values after corrections. The Microsoft Excel add-in solver was used to find the optimal values for *a*, *b*, and *c* for a least square fit for the relation:

$$0={Bc}_{n}-c\Delta {Br}_{n}-(a\;t_{n}+b)$$

The constant *c* represents the attenuation (the above scale factor) of the external field by the MSR which is dependent on the SF and the position of the reference fluxgate. The yellow line is defined by the parameters *a* and *b*. Parameter *a* is the slope of the assumed linear DC-offset drift of the measuring equipment. The average value of the yellow linear fit curve over the scan time duration is the best approximation of the field value at the MSR center for this scan (this value is nearly identical to the average value of the seven points of the blue curve). This value depends on *b* and is added after subtracting the linear fit curve to conserve this value for the final black curve. Otherwise, the 3D field maps after applying a correction with the same parameters *a*, *b*, and *c* to the 125 points *B_i_* of the scan will show the field changes with respect to the center of the MSR. The final equation after combining the different contributions for the correction is as follows:

$$B_{i,corrected}=B_{i}-c∆B_{i}-at_{i}+a\frac{t_{scan,end}-t_{scan,start}}{2}$$

If there is a single dominant disturber or the constant c is similar for the main disturbers, the correction will work nicely. It fails when one of the main disturbers has a dissimilar c factor, e.g., two disturbing sources not so far away from the MSR but one of them on the side with the outside reference detector and the other in the direction of the opposing MSR wall. In our case for all three mappings, the remaining field changes in the vector components are on the order of 0.3 nT (about 0.2 for the black curve in Figure A1).

We tried fits with identical *c* factors in the x, y, and z direction, respectively, for the three field scans, or allowing individual *c* factors for the three scans. The results are very similar, which we expected because the position of the reference detector was not changed. We therefore used the identical *c* factors *c_x_* = 0.0080, *c_y_* = 0.0246, and *c_z_* = 0.0105 for the corrections. The expectation is that 1/c is in the range of the low-frequency shielding factor of 100 with deviations for the different directions since the reference detector is at one side and within 1.5 m distance of the MSR.

An overview of the optimized correction applied to the seven repeated measurements of the field at the center of the MSR is shown in Table A1.

**Table A1:** Statistics when the optimized correction is applied to the 7 repeated measurements of the field at the center of the MSR.

Values	posI ΔB_center_ Corrected-Measured				negI ΔB_center_ Corrected-Measured				posZ ΔB_center_ Corrected-Measured			
in nT	ΔBx	ΔBy	ΔBz	Δ|B|	ΔBx	ΔBy	ΔBz	Δ|B|	ΔBx	ΔBy	ΔBz	Δ|B|
**min**	−0.30	−0.15	−0.43	−0.14	−0.13	−0.36	−0.48	−0.42	−0.23	−0.40	−0.18	−0.13
**max**	0.36	0.74	0.23	0.07	0.09	0.65	0.26	0.26	0.17	0.51	0.31	0.12
**range**	0.66	0.89	0.66	0.21	0.22	1.00	0.74	0.67	0.40	0.91	0.49	0.25
**mean**	0.08	0.04	−0.01	−0.02	−0.01	0.02	−0.03	−0.06	0.02	−0.03	0.10	0.03
**STD**	0.236	0.315	0.232	0.086	0.086	0.371	0.287	0.256	0.152	0.384	0.186	0.100

## Appendix B. Curly Residual Fields Caused by the Choice of the Demagnetization Function

The concept of curl, specifically a magnetic field surrounding the MSR center, is grounded in the behavior of magnetic domains within the shielding material. If the magnetic domains’ orientations are randomly distributed, the shielding wall exhibits minimal magnetic field externally. This holds true even for a local 50:50 distribution aligned with the degaussing field, as shown by the blue arrows in Figure 1. However, any deviation from this 50:50 distribution results in a residual magnetic field aligned with the degaussing direction. This residual field manifests as a circular magnetic field inside the MSR, with its amplitude increasing closer to the MSR walls.

A potential cause for the development of a curl is a DC offset in the applied field. This offset consistently leaves a few more magnetic domains aligned in one direction during each degaussing cycle.

Another factor that can lead to the development of a curl is an insufficient number of degaussing cycles. Theoretically, an infinite number of cycles would achieve a perfect 50:50 distribution of magnetic domains, provided there are no DC offsets. However, increasing the number of cycles also increases the time required for degaussing. If too few cycles are used, a circular residual field may emerge because the precision of the 50:50 distribution diminishes with fewer active cycles. Active cycles are those that do not exceed the saturation point within the material. In passive cycles, the direction of all domain fields flips every half cycle, whereas, in active cycles, a small number of domains remain oriented in opposite directions after each half cycle.

At the start of degaussing, the current must be sufficiently strong to saturate the entire shielding material of the walls being degaussed. Throughout the degaussing process, the field generated in the shielding wall by the current in the degaussing coil is not uniform, being particularly stronger near the coil windings. In our setup, these windings are located at the edges of the cubical MSR.

For both **posI** and **negI** configurations, sequential degaussing was performed in the order of I_y_-coil, I_x_-coil, and then I_z_-coil. Based on our experience, it is most effective to start by degaussing the four walls surrounding the wall with the door using the I_y_-coil, followed by the four walls where the magnetic flux is parallel to the long side of the door opening, which is achieved with the I_x_-coil. Finally, the I_z_-coil is used to generate a flux as indicated by the blue arrows in Figure 1. Although there is no known theory explaining why this sequence is optimal, it is our preferred method. By using the I_z_-coil last, we anticipate the strongest circular field distributions along the four vertical edges of the cubical 3D field map. In the **posZ** configuration, connecting all I_x_-, I_y_-, and I_z_-coils in series creates a magnetic flux around one of the four possible diagonals between two opposing corners, all intersecting the center of the MSR. The specific diagonal depends on how the coils are connected relative to each other. For **posZ**, the circular field distribution is expected to concentrate around one of these diagonal lines, being most intense at the two opposing corners.
